# Profiles of suicide attempted in children and adolescents

**DOI:** 10.1016/j.jped.2024.01.007

**Published:** 2024-04-11

**Authors:** Alicia Ortega-Narváez, Diana Marcela Muñoz-Manquillo, Claudia Patricia Guzmán-Lopez, Ginna Cabra-Bautista

**Affiliations:** a Universidad del Cauca, Department of Pediatrics, Popayán, Colombia; b Hospital Susana López de Valencia, Pediatric Emergency, Popayán, Colombia; c Hospital San José, Pediatric Emergency, Popayán, Colombia

**Keywords:** Suicide, attempted, Suicide, Child, Adolescent, Armed conflicts

## Abstract

**Objective:**

Suicide attempt (SA) is the strongest predictive variable for completed suicide. The Department of Cauca in Colombia has an SA rate higher than the national average, but the factors are unknown. The objective was to identify the profiles of SA in children and adolescents of Cauca.

**Methods:**

Cross-sectional study, which included all SA (Event-356) records from the SIVIGILA platform in children under 18 years of age between 2016 and 2019. The authors described the variables and multiple correspondence analysis (MCA) with the Burt method, according to the completeness of the data to establish the possible SA profiles using STATA 15.1, and R. The Ethics Committee at Universidad del Cauca approved it.

**Results:**

The study found 977 SA during this period, 72.4% female, 97.1% adolescent, 74.4% mestizo, 19.3% indigenous, 45.3% resided in municipalities exposed to the armed conflict, 32.3% expressed ideation and previous attempts, and 15.5% prior attempts. The MCA included 810 SA and identified three profiles: “Classic”, which had mestizo adolescents with a history of prior SA, mental illness, or psychoactive substance use problems; “Related to the armed conflict”, which included female adolescents with a first SA and residents in municipalities exposed to the armed conflict; “Ethnic” represented by male indigenous, with housing in a rural area.

**Conclusion:**

The SA profiles found in Cauca were “Classic”, “Related to the armed conflict”, and “Ethnic”; these can be considered to implement prevention strategies from a cross-cultural, mental health, and gender perspective, with the presence of the state in the territories.

## Introduction

Suicide has been classified as a public health problem with an average global rate of nine suicides per 100,000 inhabitants in 2019.^1^ It mainly affects young people between 15 and 19 years of age for whom it is the fourth cause of death.^1^ In Colombia, according to data from the National Administrative Statistics Department, the suicide rate has been on the rise, becoming the second cause of death in individuals between 10 and 24 years of age; in 2015, 14% of the national total of suicides occurred in the pediatric population.^2^ Due to these increases, Colombia began epidemiological surveillance of Suicide Attempt (SA) in 2016.^3^

The department of Cauca, located in southwestern Colombia, has a population of nearly 1,500,000 inhabitants, of which, almost 63% live in rural areas; ^4^ Its demographic profile includes a wide ethnic diversity with mestizo, afro-Colombian, and indigenous populations; it has been one of the departments most exposed to armed conflict throughout the country's history.^5^ The Colombian State categorized the municipalities of the country according to the effects of the internal armed conflict on its civilian population. In this way, those with the greatest armed confrontation and victimization factors (homicides, kidnappings, looting, displacements, land mines, forced disappearances and murder of union workers, local authorities, journalists and land claimants) were prioritized.^5^

Cauca has an SA rate higher than the reported national average, and the population factors that could explain this higher frequency are unknown.^6^ The previous SA is the major individual predictive variable of death by suicide in the general population, and the frequency of SA is 20 times higher than suicide.^7^^,^^8^ Other risk factors have also been described, such as cultural and social influences, age, gender, stressful life events, and predisposing pathologies.^2^^,^^8^^,^^9^ This occurrence generates a psychological and emotional effect on the individual, the family, and the community, in addition to a significant impact on health services, a situation that must be identified and managed early. The objective of this study was to identify the SA profiles in children and adolescents from the Department of Cauca.

## Methods

A cross-sectional study was carried out with information about SA (identified in the registers with the code Event 356) provided by the Sistema Nacional de Vigilancia en Salud Pública (SIVIGILA) for the systematic registration of important events in public health.^10^ It is filled out when a person seeks medical attention at any level of care in the country for injuries associated with SA. This register is mandatory in both the public and private sectors. The study included all SA records in children under 18 years between 2016 and 2019 and excluded duplicate registries and non-resident children or adolescents in the Department of Cauca, Colombia.

The work described the variables contained in the SA notification form (Event 356) that provide information regarding sociodemographic characteristics, presence of previous SA, risk factors, type of substance or suicide method used, and referral to psychiatry, psychology, or social work.^10^ The study also presented the variables with absolute frequencies and percentages.

A multiple correspondence analysis (MCA) was performed, using the Burt method with inertia adjustment. For the analysis, the authors only considered the years in which the variables to be evaluated had the best quality in terms of completeness, frequency, and clinical importance to evaluate the relationships between the categories and establish the possible SA profiles. It evaluated eight variables: age, sex, ethnicity, municipality of residence exposed or not exposed to the armed conflict, urban or rural area of residence, previous suicide attempts, history of psychiatric illness, and history of psychoactive substance use problems. The information was analyzed using the STATA 15.1 and R 4.2.0 package ca.

The study was approved by the Ethics Committee at Universidad del Cauca and registered in the information system of the research vice-rectorate with number 5478 on 10 August 2020. The study was classified as observational research without risk, according to Resolution 8430 of the Colombian standard. Data was gathered from the records of Event 356 in SIVIGILA without including information on the identity of minors.

## Results

During the study period between 2016 and 2019, 977 SA were recorded in children under 18 years of age in the department of Cauca, documenting a progressive increase. Regarding the sociodemographic characteristics, most of the SA occurred in adolescents between 12 and 17 years of age (97.1%), mainly female (72.4%), mestizo (74.4%), affiliated with the subsidized health regime (78.0%); around 20.0% with basic primary education and 23.1% without information on the educational level. About half of the children and adolescents resided in municipalities prioritized because of the Colombian internal armed post-conflict (45.3%). A third belonged to at-risk population groups, such as pregnant women, the disabled, victims of violence, displaced persons, migrants, the homeless, children in charge of the Colombian Family Welfare Institute or belonging to psychiatric centers (Table 1)**.**

**Table 1:** Characteristics of children and adolescents with suicide attempt in Cauca, *n* = 977.

Variables	n (%)
**Year SA Reported**	
2016	167 (17.1)
2017	259 (26.5)
2018	255 (26.1)
2019	296 (30.3)
**Sex**	
Male	270 (27.6)
Female	707 (72.4)
**Age**	
Childhood (< 12 years)	28 (2.9)
Adolescence (12–17 years)	949 (97.1)
**Ethnic group**	
Mestizo a	727 (74.4)
Indigenous	189 (19.3)
Afro-Colombian	61 (6.2)
**Social Security Scheme**	
Contributory	187 (19.1)
Subsidized	762 (78.0)
No health insurance	28 (2.9)
**Educational level**	
Secondary or higher	548 (56.1)
Up to primary school	198 (20.3)
Out of school	5 (0.5)
No information	226 (23.1)
**Municipality of residence**	
No exposure to the armed conflict	534 (54.7)
Exposed to the armed conflict^b^	443 (45.3)
**Area of residence**	
Urban	496 (50.8)
Rural	481 (49.2)
**Occupation**	
Does not work	852 (87.2)
Works	38 (3.9)
No information	87 (8.9)
**Population group**	
Other groups	660 (67.5)
Risk groups^c^	317 (32.5)
**Remission**	
Psychiatry	752 (76.9)
Psychology	652 (66.7)
Social work	360 (36.8)

a Mestizo: descendants of white European and Native American parents.

b Prioritized municipalities for post-conflict (Decree 893 of 2017).

c Risk groups: Pregnant women, disabled, victims of violence, displaced persons, migrants, street dwellers, in charge of the ICBF, or in psychiatric centers.

Among the risk factors reported, it was found that in those under 18 years of age, 8.7% had had suicidal ideation without previous attempts, 15.5% had had previous ideation and attempts, and 32.3% had a previous suicide attempt. Other risk factors were relationship problems, a history of psychiatric illness, and the use of psychoactive substances. Among the risk factor variables, the authors found observations with no data, and we do not know the cause of this lack of information in the registers, mainly history of psychiatric illness (58.8%) and unspecified violence (21.0%) (Table 2).

**Table 2:** Risk factors identified from the mandatory notification form of SIVIGILA Event 356 in Cauca, *n* = 977.

Risk factors	n (%)
**Previous suicide attempts**	316 (32.3)
**Suicidal ideation without prior attempts**	85 (8.7)
**Suicidal ideation with previous attempts**	151 (15.5)
**Relationship problems**	304 (31.1)
**History of chronic disease**	46 (4.7)
**Legal Problems**	27 (2.8)
**Suicide of a family member**	69 (7.1)
**Substance consumption problems**	114 (11.7)
Death of a family member*	
Yes	43 (4.4)
No information	205 (21.0)
Educational problem*	
Yes	139 (14.2)
No information	205 (21.0)
History of violence*	
Yes	30 (3.1)
No information	205 (21.0)
Labor problems*	
Yes	42 (4.3)
No information	205 (21.0)
History of psychiatric illness*	
Yes	266 (27.2)
No information	574 (58.8)

⁎ The category **No** of these variables is the remainder of n (%).

The most frequently used SA methods were suicide method used was poisoning (68.7%) and sharp weapons (21.4%). The most used substances were medications (25.0%) and pesticides (21.8%); however, no information was found on the substance used in 45.5% of the records. Of the 977 patients, 76.9% were referred to a psychiatric service, 66.7% to psychology, and 36.8% to social work (Table 1).

Due to a significant percentage of incomplete data in the registry for 2016, the year the notification sheet was implemented in Colombia, the multiple correspondence analysis (MCA) only contains data from 2017 to 2019. The MCA using the Burt method included 810 SA events.

**Figure 1:**
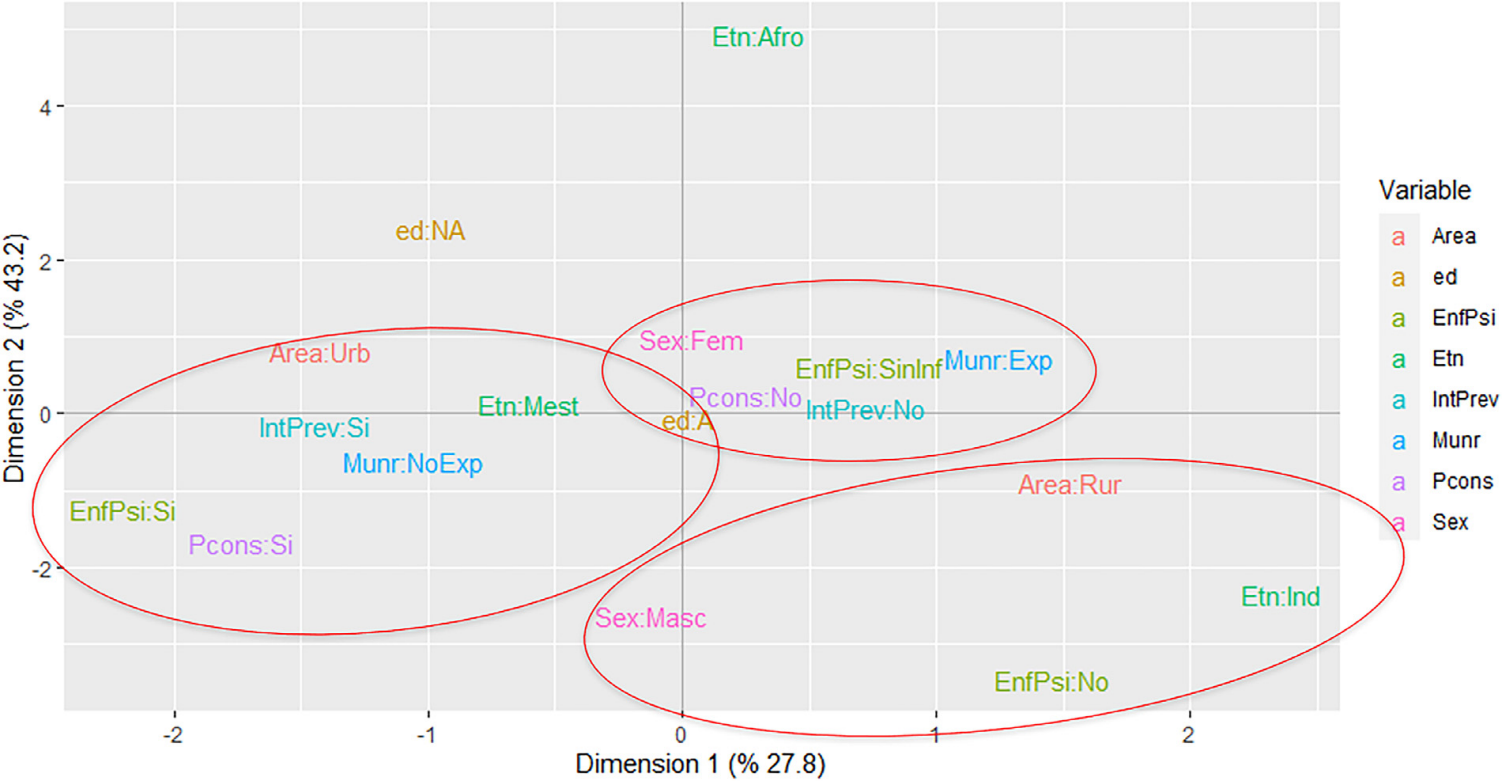
Profiles of the Suicide Attempt in children and adolescents of Cauca Colombia, 2017–2019. Variables used: int_prev (previous suicide attempt); pconsumption (history of problem with consumption of psychoactive substances or alcohol); mun_r (municipality of residence exposed to the conflict); sexum (0: male, 1: female); enfpsinum (history of psychiatric illness); age (0: childhood, 1: adolescence); area (0: Urban, 1: Rural); ethnic group (0: mestizos, 1: indigenous, 2: Afro-Colombians). Convention response 0: No, 1: Yes.

It was found that the first two dimensions of the MCA explained 71% of the data variability, and it was possible to identify three profiles denominated: “Classic”, “Related to the armed conflict”, and “Ethnic” profiles. The childhood category (children under 12 years of age) was not found in any of the profiles identified. The "Classic" profile identified mestizo adolescents living in municipalities not exposed to the armed conflict, with a history of previous suicide attempts, a history of some mental illness, or problems with consumption of psychoactive substances. The profile "Related to the armed conflict" identified female adolescents with a first SA who lived in municipalities exposed to the armed conflict without information on a history of psychiatric illness or history of problems with the use of psychoactive substances. The study identified a possible "Ethnic" profile, which found an approximation of the indigenous categories, male, living in a rural area, and without a history of mental illness. The authors present a self-made image for each profile found and we credit the illustrator of the images (Figure 1).

## Discussion

A suicide attempt is a public health problem that becomes a warning condition due to its relationship with multiple personal, family, and social issues; it must be recognized, monitored, and intervened opportunely.^2^^,^^6^^,^^7^ This population study used an exploratory technique to analyze categorical data that allowed the identification of three SA profiles in children and adolescents in a Colombian region that is demographically diverse and affected by critical social problems.^2^^,^^7^

**Figure 2:**
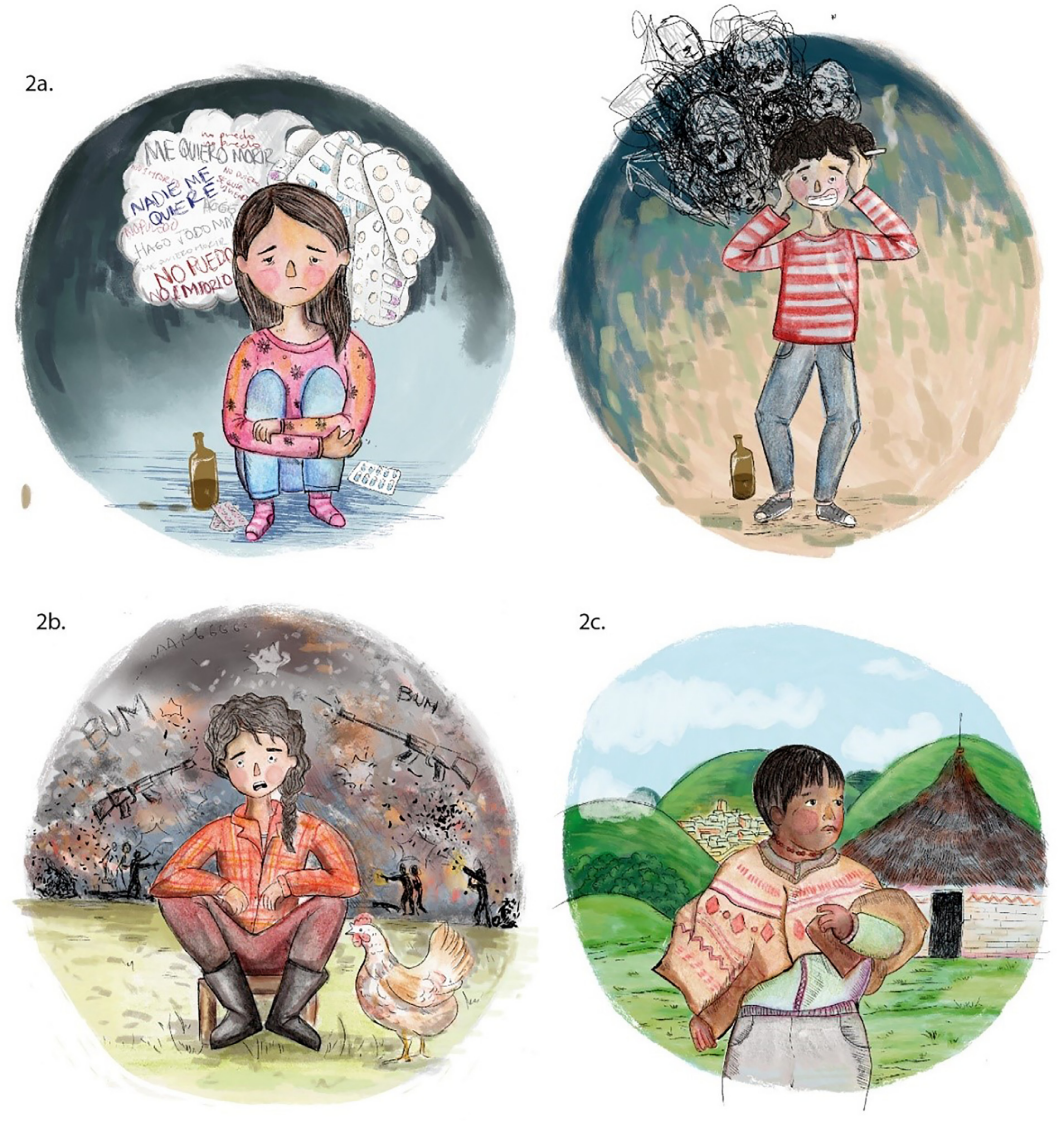
Profiles of the Suicide Attempt in children and adolescents. (2a) “Classic” Profile. (2b) “Related to the armed conflict”. (2c) “Ethnic” Profile.

The "Classic" profile includes mixed-race adolescents with a history of previous SA, some mental illness, or use of psychoactive substances. This name was assigned by the authors because it agrees with the most-often reported profile in the literature (Figure 2a). It has been reported that a previous SA is the leading independent risk factor for suicide and that the principal associated mental illnesses are depression, bipolar disorder, and anxiety.^2^^,^^6^^,^^11^ Similarly, the consumption of psychoactive substances is related to suicidal behavior and triples the risk of suicide related to depressed mood.^2^^,^^12^, ^13^, ^14^

The profile "Related to the armed conflict" found female adolescents with a first SA living in municipalities with a history of exposure to the armed conflict (Figure 2b). The department of Cauca has been one of the most affected by this phenomenon, with 20 of 42 towns prioritized for the Colombian armed post-conflict.^15^ The literature describes a higher frequency of SA in people exposed to armed conflict, military population, and adults displaced by violence.^16^, ^17^, ^18^ In the child and adolescent population, information is scarce, although, in Middle Eastern countries, a higher frequency of depression and suicidal ideation is reported in those exposed to terrorist attacks.^19^ A Colombian study found a prevalence of major depression close to 50% and suicidal ideation of 34.5% in children under 13 years of age exposed to armed conflict.^20^ The authors believe that the predominant female sex in this profile corresponds to a probable situation of gender violence in populations with a greater situation of conflict, in this case armed conflict.^21^

The "Ethnic" profile included indigenous males residing in rural areas (Figure 2c) and coincides with the descriptions of other studies on native populations of Australia,^22^ the United States,^23^ Norway,^24^ Panama,^25^ and Brazil ^26^ where suicide rates are higher in indigenous populations compared to non-indigenous ^27^ and higher in developing countries, which seems to reflect the social inequity in these populations.^28^ In other Colombian regions, such as Vaupés, Córdoba, and Chocó, conflictive relationships were identified in the indigenous family environment due to ethnocultural situations with acculturation/deculturation phenomena and the absence of professional support due to mental health problems.^29^^,^^30^

The study's principal strength was the use of population-based mandatory reporting registries that explored SA in the child and adolescent population of a multi-ethnic region exposed to significant territorial and economic deprivation. These findings may have external validity in regions of the world with similar characteristics to ours.

Limitations were evident with the quality of the records because they are secondary data and do not depend on the authors of this work; this implied possible information bias despite strategies to minimize this impact. Mandatory reporting of SA is done from any level of health care, however, those who have an SA and do not consult will not be included in the national registers.

Another limitation is that the variables contemplated in the SA notification form do not allow for adequate differentiation between risk and precipitating factors, and several factors related to SA, such as bullying, types of violence, and other relevant mental illnesses, are included but not differentiated.

The study does not include notifications from 2020 because the Covid-19 pandemic is likely related to different factors not comparable with previous years. For future implications, research should involve evaluation during and after the pandemic and designs that allow delving into the factors not included in the mandatory notification form. Furthermore, future studies may relate consummated suicides to the suicide attempt profiles found.

## Conclusion

In Cauca, a region of the Colombian Pacific, three profiles were identified of suicide attempts in children under 18 years of age. A Classic profile with a previous suicide attempt, history of mental illness or consumption of psychoactive substances; a profile Related to the Armed Conflict in adolescent women with a first SA living in municipalities with a history of strong exposure to the armed conflict; and a third Ethnic profile in indigenous male adolescents. These findings must be considered to implement prevention strategies from a cross-cultural, mental health, and gender perspective with the state's participation in the territories.

## Conflicts of interest

The authors declare no conflicts of interest.
